# Characteristics of Umami Taste of Soy Sauce Using Electronic Tongue, Amino Acid Analyzer, and MALDI−TOF MS

**DOI:** 10.3390/foods13142242

**Published:** 2024-07-16

**Authors:** Ting Cai, Nan Hai, Peng Guo, Zhi Feng, Yu Zhang, Jing Wang, Zhipeng Yu, Huan Liu, Long Ding

**Affiliations:** 1 College of Food Science and Engineering, Northwest A&F University, Xianyang 712100, China; cting3399@nwafu.edu.cn (T.C.); hainan@nwafu.edu.cn (N.H.); gpeng815@nwafu.edu.cn (P.G.); fengzhiwow@nwafu.edu.cn (Z.F.); xnzy@nwafu.edu.cn (Y.Z.); wj6746@nwafu.edu.cn (J.W.); 2 College of Food Science and Engineering, Hainan University, Haikou 570228, China; yuzhipeng20086@sina.com; 3 Chongqing Institute for Food and Drug Control, Chongqing 401121, China; liuh985@163.com; 4 Key Laboratory of Condiment Supervision Technology for State Market Regulation, Chongqing 401121, China

**Keywords:** soy sauce peptide, umami, electronic tongue, amino acid, MALDI−TOF MS

## Abstract

The objective of this study was to investigate the umami characteristics of soy sauce using electronic tongue evaluation and amino acid composition and matrix-assisted laser desorption ionization–time of flight mass spectrometry (MALDI−TOF MS) analysis. The soy sauce peptides were isolated from soy sauce using XAD−16 macroporous resin combined with ethanol solution. The results showed that the soy sauce peptide fraction eluted by 60% ethanol (SS−60%) exhibited a prominent umami taste, and the umami scores were highly positively correlated with the amino acid nitrogen contents of soy sauces. The umami scores of SS−60% were significantly positively correlated with the contents of free amino acids. Especially, Phe showed the highest positive correlation with the umami scores. In addition, five characteristic ion peaks with *m*/*z* at 499, 561, 643, 649, and 855 were identified in the peptide mass fingerprinting. Therefore, this study provides new insights into the umami characteristics for the taste evaluation and reality identification of soy sauce.

## 1. Introduction

Soy sauce is a traditional condiment with a long history of more than 5000 years. It is produced primarily from soybeans and wheat under the fermentation of microorganisms, including *Aspergillus oryzae*, *Aspergillus sojae*, etc. [1,2,3]. Soy sauce not only has a unique color, aroma, taste, and nutritional value but also has the effect of harmonizing food taste, improving food color, and removing the fishy taste of food. Therefore, soy sauce has become an indispensable seasoning all over the world [4,5,6]. Fermentation plays a critical role in forming the unique taste of soy sauce [7]. The traditional fermentation of soy sauce is mainly natural sunbaked night dew fermentation and salt-free solid-state fermentation. However, with the development of fermentation technology, high-salt liquid fermentation and low-salt solid fermentation are mostly used now [8]. Compared with low-salt solid-state fermented soy sauce with high production efficiency and low cost [9], high-salt liquid fermented soy sauce has a strong taste and better quality. Therefore, high-salt liquid fermented soy sauce is preferred by consumers [10].

The taste of soy sauce generally consists of umami, salty, sweet, sour, bitter, and kokumi [11]. Although the overall taste of soy sauce is a result of the balance of the various tastes, umami is the most important taste [12]. What should be noted is that the taste of soy sauces varies greatly due to the raw materials, microorganisms, and manufacturing technologies used [13]. It is generally considered that the quality of soy sauce depends on the amino acid nitrogen content. However, the major components that contribute to the taste, especially the umami, of soy sauce and the characteristics of the umami taste of soy sauces with different amino acid nitrogen contents are not well known.

Umami, known as the fifth taste, makes the overall taste of food softer, more harmonious, and full-bodied, helping improve the overall sensory characteristics of food [14]. It is known that the umami taste of soy sauce is highly associated with amino acids, peptides, nucleotides, and other taste substances [15]. Especially, it is generally believed that the free amino acids of Glu, Asp, Phe, Ala, Gly, and Tyr have umami taste or umami-enhancing effects [16,17]. In addition, some gamma-glutamyl peptides and the peptides containing umami amino acids such as Glu, Asp, and Gly produced from soy protein by fermentation have been found with umami tastes and play important roles in the unique taste of soy sauce [15,18,19]. Consequently, the interest in discovering novel umami peptides from soy sauce and other food sources is rising. For instance, the short peptides γ-Glu-Glu, Glu-Glu, γ-Glu-Cys, γ-Glu-Leu, Glu-Leu, and Ile-Glu were demonstrated to exhibit umami taste [17]. The peptides LPEEV, AQALQAQA, EQQQQ, ALPEEV, and EAGIQ that were identified from soy sauce were also determined to show umami taste and umami-enhancing effects [20]. Nevertheless, the amino acid and peptide profiles underlying the umami taste of soy sauce with different amino acid nitrogen contents have not been reported.

Therefore, the purpose of this study was to elucidate the umami characteristics of soy sauces with different amino acid nitrogen contents. The umami peptides from soy sauces were first isolated using XAD−16 macroporous resin adsorption combined with ethanol elution. Then, the umami characteristics of the soy sauce peptides were analyzed by electronic tongue, amino acid analyzer, and matrix-assisted laser desorption ionization–time of flight mass spectrometry (MALDI−TOF MS).

## 2. Materials and Methods

### 2.1. Materials and Chemicals

Twelve kinds of brewed soy sauces were purchased from a local supermarket in Yangling, Shaanxi, China, and stored at room temperature. The fermentation technology, amino acid nitrogen content, and raw materials of soy sauce are shown in Table S1. The XAD−16 macroporous resin was purchased from Aladdin Biochemical Technology Co., Ltd. (Shanghai, China). All other chemicals were of analytical grade.

### 2.2. Isolation of Soy Sauce Peptides by XAD−16 Macroporous Resin

Before the experiment, the monomers and chemical reagents were removed from the XAD−16 macroporous resin according to the previous method [21]. The XAD−16 macroporous resin was soaked in 95% (*v*/*v*) ethanol for 24 h followed by 5% (*m*/*v*) NaOH for 5 h and then 5% (*v*/*v*) HCl for another 5 h. Then, the XAD−16 macroporous resin was washed using distilled water and dried at 60°C for 48 h in an oven (DHG-9240A, Shanghai Jing Hong Experimental Equipment Co., Ltd., Shanghai, China) and stored at room temperature until use.

The soy sauce peptides were isolated using XAD−16 macroporous resin referring to the previous method with slight modifications [20]. Briefly, 5 g of the XAD−16 macroporous resin was immersed in 95% ethanol for 12 h for activation. After the resin was washed using distilled water until the ethanol was completely removed, 100 mL of the soy sauce was thoroughly mixed with the resin at 30°C for 10 h in an incubator with constant shaking at 150 r/min. The soy sauce peptides that adsorbed on the resin were first eluted by adding 300 mL of distilled water and incubated at 30°C for 12 h in an incubator with constant shaking at 150 r/min. The eluent (SS−0%) was subsequently collected using a stainless-steel cell strainer (100 Screen Mesh, Solarbio Science & Technology Co. Ltd., Beijing, China). Then, the soy sauce peptides that adsorbed on the resin were sequentially eluted with 300 mL of 20% (*v*/*v*) ethanol, 40% (*v*/*v*) ethanol, 60% (*v*/*v*) ethanol, and 80% (*v*/*v*) ethanol at 30 °C for 12 h in an incubator with shaking at 150 r/min, respectively. The eluents were collected as SS−20%, SS−40%, SS−60%, and SS−80%, respectively. All the soy sauce peptide fractions (SS−0%, SS−20%, SS−40%, SS−60%, and SS−80%) were lyophilized and stored at −20°C until further analysis.

### 2.3. Electronic Tongue Analysis

The differences in the taste characteristics of soy sauce peptides were analyzed using the Astree II electronic tongue system equipped with seven sensors (ZZ, JE, BB, CA, GA, HA, and JB) (Alpha MOS, Toulouse, France) according to the described method with a slight modification [22]. Before the experiment, the electronic tongue system was calibrated and diagnosed using distilled water, 0.01 mol/L HCl, 0.01 mol/L NaCl, and 0.01 mol/L monosodium glutamate (MSG), respectively. The soy sauce peptides were diluted to 0.1 mg/mL using deionized water and then filtered using a Millex*^®^* PVDF needle filter (0.45 μm). Then, 85 mL of the soy sauce peptide solution was analyzed, and the taste profile was measured with seven replications, and the last four data were selected for analysis using the PCA method.

Furthermore, the taste scores of soy sauce peptides, including sourness, bitterness, astringency, aftertaste-B (bitter aftertaste), aftertaste-A (astringent aftertaste), richness, umami, and saltiness, were analyzed using the SA402B electronic tongue (INSENT, Atsugi City, Japan) according to the previous method [23]. Briefly, 10 mg of the sample was dissolved in 100 mL deionized water and then sequentially filtered using 0.45 µm and 0.22 µm Millex*^®^* PVDF needle filters (Millipore, MA, USA). The sample solution was then taken into a beaker and tested four times using the SA402B electronic tongue. The procedure was set to 30 s for taste collection, aftertaste collection time of 30 s, and cleaning time of 300 s. The last three data were collected for analysis.

### 2.4. Determination of Amino Acid Composition

The determination of the amino acid composition of soy sauce peptides was performed according to the previous method with some modifications [24,25]. The sample was dissolved in distilled water at 5 mg/mL and placed at room temperature for 30 min. Then, the sample solution was mixed with 10% sulfosalicylic acid at a volume ratio of 4:1 (*v*/*v*) and incubated at 4 °C for 60 min for deproteinization. After centrifugation at 10,000 r/min and 4°C for 15 min, the supernatant was collected and filtered with a 0.22 μm Millex^®^ PVDF needle filter. For the free amino acids, the obtained supernatant was directly analyzed using an automatic amino acid analyzer (A300 advanced, Membrapure, Germany). For the hydrolyzed amino acids, the sample was first hydrolyzed with 10 mL 6 M HCl at 110°C for 24 h and then deacidified by nitrogen blowing at 70 °C before the analysis.

### 2.5. MALDI−TOF MS Analysis

The soy sauce peptides were determined by MALDI−TOF MS according to the method of Wang et al., with some modifications [26]. Briefly, the sample was dissolved in the matrix solution (MeOH) (2 mg/mL) and subsequently mixed with 4-cyano-α-hydroxy cinnamic acid (CHCA) (1 mg/mL) before being applied to the metal target plate. After drying, the MALDI−TOF MS technique employs air as a collision gas for the high-resolution determination of samples in positive mode, utilizing 20 kV collision-induced dissociation. The sample and matrix undergo sublimation and ionization through MALDI. Subsequently, the resulting ions were separated based on their *m*/*z* values using a TOF analyzer (Autoflex, Bruker, Germany), while MS software (version 3.4 Bruker Daltonics, Billerica, MA, USA) generated and analyzed the spectral characteristics of these ions, ultimately yielding an MS spectrogram.

### 2.6. Statistical Analysis

All data were presented as the mean ± standard deviation and analyzed using Origin 9.5 software (Origin Lab Corporation, Northampton, MA, USA). Origin 9.5 software was also used for Pearson’s correlation analysis. The difference was carried out by one-way analysis of variance (ANOVA) followed by Duncan’s test with a significance value of *p* < 0.05 by SPSS 26 software (IBM, Chicago, IL, USA).

## 3. Results and Discussion

### 3.1. Taste Characteristics of Soy Sauce Peptides by Astree II Electronic Tongue

**Figure 1:**
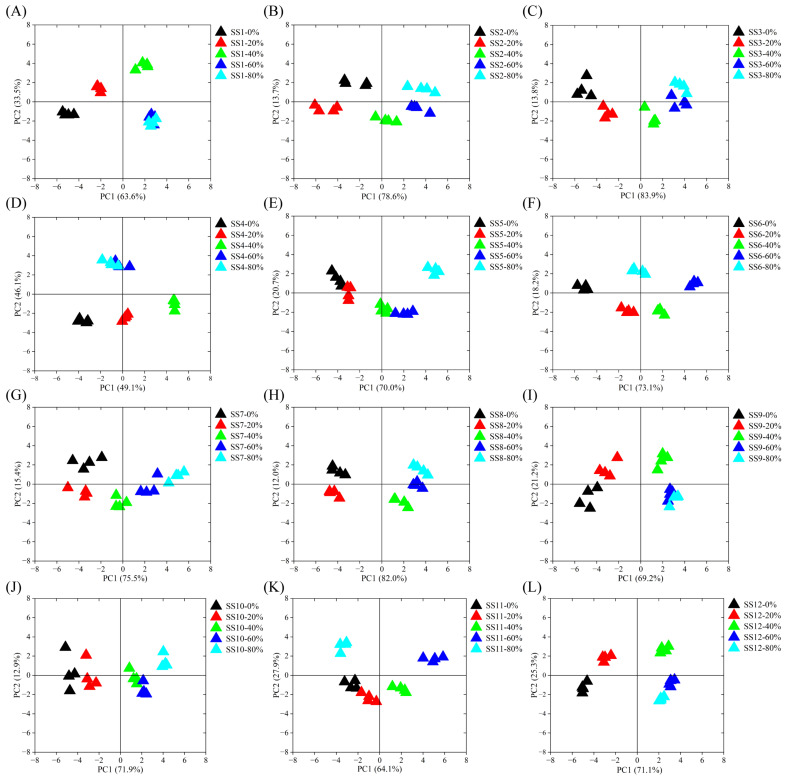
PCA of the taste properties of soy sauce peptide fractions eluted by ethanol with different concentrations by electronic tongue evaluation. (**A**) SS1; (**B**) SS2; (**C**) SS3; (**D**) SS4; (**E**) SS5; (**F**) SS6; (**G**) SS7; (**H**) SS8; (**I**) SS9; (**J**) SS10; (**K**) SS11; (**L**) SS12.

Macroporous resins are usually used to isolate bioactive components from natural products due to their large specific surface area, optimal pore structure, and unique adsorption properties [27]. It had been confirmed that XAD−16 macroporous resin was appropriate for the purification and enrichment of soy sauce peptides with high adsorption and desorption capacities [28,29]. In this study, the soy sauce peptides were first adsorbed using XAD−16 macroporous resin and then sequentially eluted by 0%, 20%, 40%, 60%, and 80% ethanol solution. The taste profiles of the soy sauce peptide fractions were evaluated using Astree II electronic tongue, as shown in Figure 1. Four repeated data points of SS−0%, SS−20%, SS−40%, SS−60%, and SS−80% of all soy sauce were aggregated into an independent population, indicating that the experimental data had a high repeatability. The cumulative contribution rate of PC1 and PC2 exceeded 80%, indicating that PC1 and PC2 reflected the taste characteristics of the soy sauce peptide fractions well. In the plot, when the Euclidean distance of two samples was small, the taste was likely to be identified as similar [30]. It was found that the taste profiles of the soy sauce peptide fractions SS−0%, SS−20%, SS−40%, SS−60%, and SS−80% were almost all separated from each other, and overlap was only observed between the SS−60% and SS−80% fractions of SS1, SS4, and SS9 in the PCA plots, indicating clear differences in the overall taste.

Ethanol has a very low polarity compared to water. When the eluent varies from water to ethanol, the resin environment is changed from polar to weak-polar, thus causing the desorption of amino acids and peptides of different polarity. Zhuang et al. explored the desorption rate of peptides adsorbed on the resin by ethanol with different concentrations and the results showed that the desorption rate of 80% ethanol was the lowest, and the desorption rate of 0, 20%, 40%, and 60% ethanol increased gradually [28]. Similarly, the results of Yang et al. showed that after gradient elution on the XAD−16 resin column, free phenolics were most enriched in 50% ethanol, while the least were enriched in 90% ethanol [29]. In the present study, the soy sauce peptides were mainly eluted by less than 60% ethanol solution, while the 80% ethanol had a very low yield of soy sauce peptides. Therefore, the soy sauce peptide fractions SS−0%, SS−20%, SS−40%, and SS−60% were selected for further analysis.

### 3.2. Taste Scores of Soy Sauce Peptides by SA402B Electronic Tongue

SA402B series taste analysis system comprises an artificial lipid membrane sensor that can mimic the human tongue taste. It is widely used in the objective and digital evaluation of the basic taste sensory indexes such as bitter, astringent, sour, salty, and umami taste of food [31]. The tasteless point for different tastes of the SA204B electronic tongue was 0, except for sourness (−13), and saltiness (−6) [32]. Taste scores above the tasteless point are generally considered to be meaningful, while scores below the tasteless point are considered tasteless compared to the reference liquid. In the present study, the radar plots of the taste scores of the soy sauce peptide fractions SS−0%, SS−20%, SS−40%, and SS−60% were as shown in Figure S1. The tastes, including sourness, saltiness, and astringency, were under their tasteless points, indicating that soy sauce peptides were tasteless in terms of sourness, saltiness, and astringency. However, it was interesting to find that the soy sauce peptide fractions SS−0%, SS−20%, SS−40%, and SS−60% showed significant differences in umami taste but not in bitterness, aftertaste-B, aftertaste-A, or richness.

**Figure 2:**
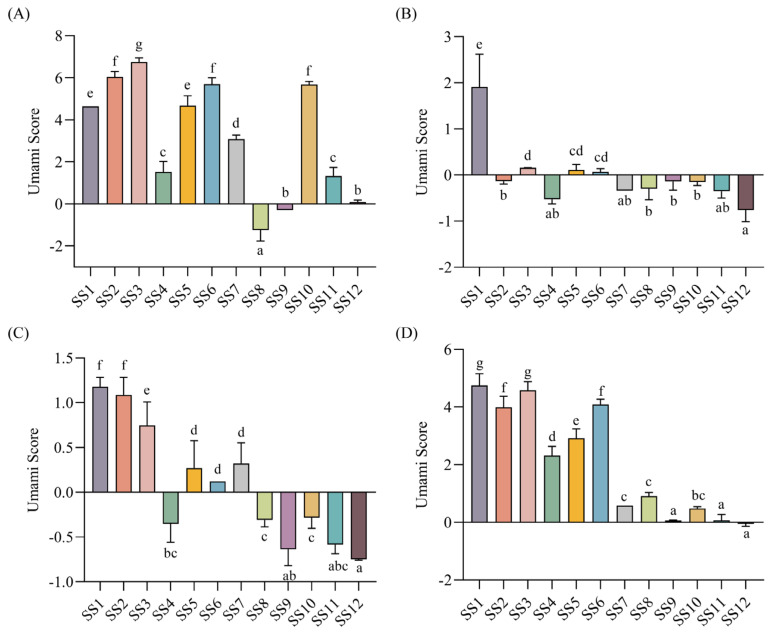
The umami scores soy sauce peptide fractions. (**A**) SS−0%; (**B**) SS−20%; (**C**) SS−40%; (**D**) SS−60%. Different letters indicated significant differences (*p* < 0.05).

The umami taste is the most important taste of soy sauce [32]. Thus, the umami scores of soy sauce peptide fractions were further analyzed. The results showed that for most of the soy sauces, the umami scores of SS−0% and SS−60% were generally higher than those of SS−20% and SS−40% (Figure S2). In addition, the umami intensities of soy sauce peptide fractions eluted with the same concentration of ethanol from 12 soy sauces were compared as shown in Figure 2. It was obvious to find that most of the peptide fractions eluted with 20% ethanol and half of the peptide fractions eluted with 40% ethanol did not show significant umami taste with the scores below the tasteless point. In contrast, most of the peptide fractions eluted with 0% and 60% ethanol exhibited a prominent umami taste.

**Figure 3:**
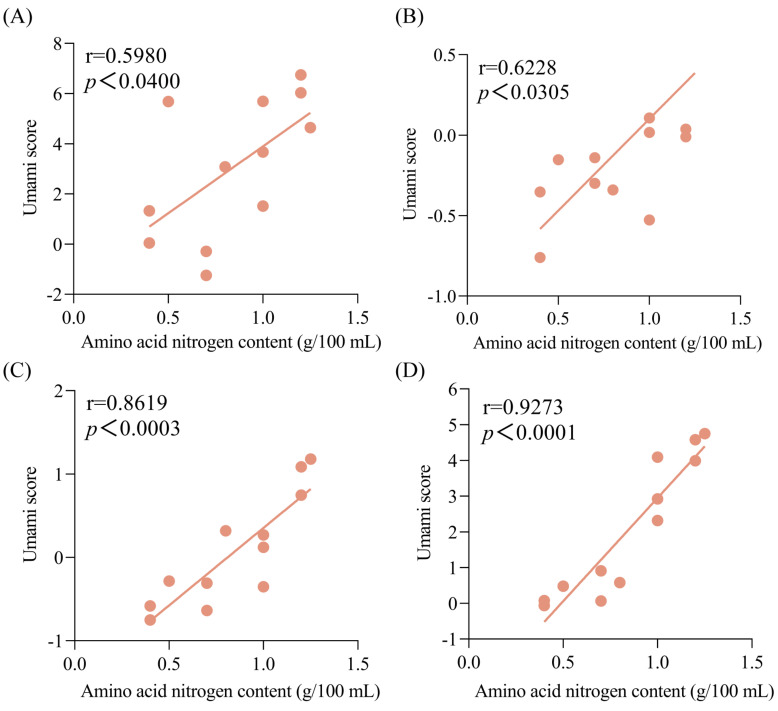
Correlations between the umami scores of soy sauce peptide fractions and the amino acid nitrogen contents of soy sauce. (**A**) SS−0%; (**B**) SS−20%; (**C**) SS−40%; (**D**) SS−60%.

Furthermore, the correlation between the umami intensities of soy sauce peptide fractions and the amino acid nitrogen contents of soy sauce was analyzed as shown in Figure 3. It was found that the umami scores of the peptide fractions SS−60% were positively correlated with the amino acid nitrogen contents of soy sauces with the highest coefficient value (r = 0.9273, *p* < 0.0001) compared with the peptide fractions SS−0% (r = 0.5980, *p* < 0.04), SS−20% (r = 0.6228, *p* < 0.0305), and SS−40% (r = 0.8619, *p* < 0.0003). This suggested that the soy sauce peptide fraction SS−60% might reflect the umami characteristics of soy sauce well and could be used in the quality evaluation of soy sauce.

### 3.3. Amino Acid Composition of Soy Sauce Peptide Fraction SS−60%

**Figure 4:**
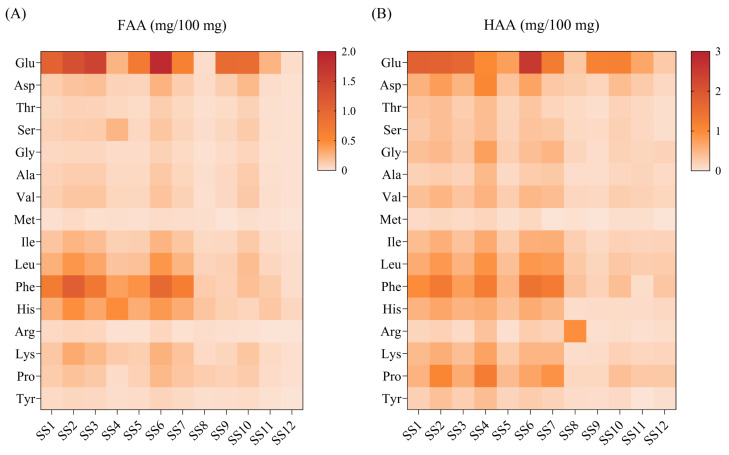
Heat map of the amino acid contents of soy sauce peptide fraction SS−60%. (**A**) Free amino acid (FAA); (**B**) hydrolyzed amino acid (HAA).

Amino acids in soy sauce are mainly derived from the hydrolysis of soy protein by fermentation and are vital for umami taste [33]. The FAAs and HAAs of 12 kinds of soy sauce peptide fractions SS−60% are presented in Figure 4. A total of 16 amino acids were detected. The total FAAs and HAAs of the 12 kinds of soy sauce peptide fraction SS−60% ranged from 0.270 to 5.544 mg/100 mg (Table S2) and 2.299 to 10.799 mg/100 mg (Table S3), respectively. Among these amino acids, Glu had the highest contents, while Met showed the lowest level. As expected, the soy sauce peptide fractions all showed a high proportion of umami amino acids (UAAs), including Glu, Asp, Phe, Tyr, Ala, and Gly.

It was interesting to find that the peptide fractions SS−60% from SS1, SS2, SS3, SS4, SS5, SS6, and SS7 showed obvious higher levels of FAAs and HAAs than that of others (Figure 4). This result was consistent with the fact that SS1 to SS7 all had amino acid nitrogen contents ranging from 0.8 g/100 mL to 1.25 g/100 mL, while the others showed amino acid nitrogen contents less than 0.8 g/100 mL. In general, the content of Glu accounted for 18–25% of the total FAAs in brewed soy sauce [34]. However, in our present study, most of the peptide fractions SS−60% from 12 kinds of soy sauces had excessive Glu levels of over 25% of the total FAAs (Table S4). In contrast, most of the peptide fractions SS−60% showed normal Glu levels less than 25% of the total HAAs (Table S5). This might be due to the addition of sodium glutamate in the production process. Thus, it was necessary to analyze the other 15 amino acid contents excluding Glu. It was found that the peptide fractions SS−60% from SS1 to SS7 showed the highest Phe, followed by His, Leu, and Ile for FAAs and followed by Leu, Pro, Leu, His, Ile, and Asp for HAAs. It was speculated that the amino acid composition might be used as an alternative indicator to the amino acid content to evaluate the quality and umami taste of soy sauces.

### 3.4. Correlation of Amino Acid Contents with Umami Taste of Soy Sauce Peptide Fraction SS−60%

**Figure 5:**
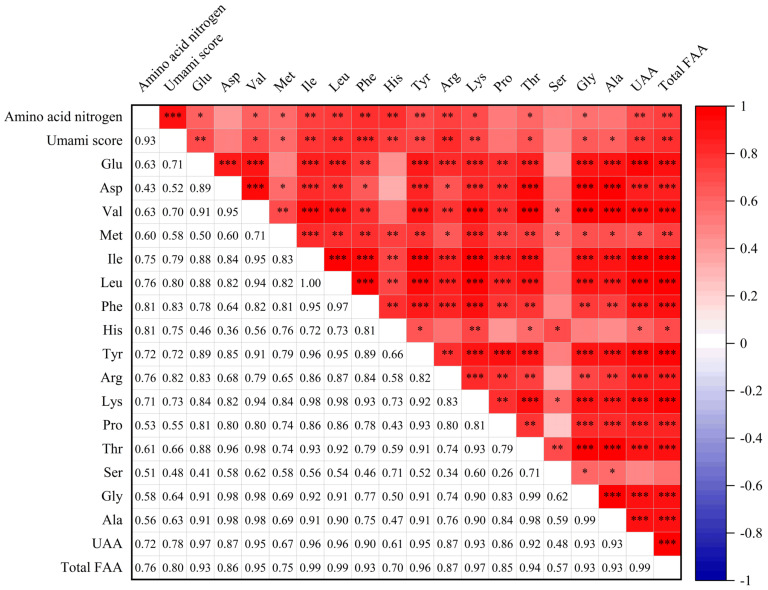
Pearson’s correlation analysis between the umami scores and free amino acid (FAA) contents of soy sauce peptide fraction SS−60%. UAAs: umami amino acids (Glu, Asp, Phe, Tyr, Ala, Gly). *** *p* < 0.001; ** *p* < 0.01; * *p* < 0.05.

To explore the relationship between the amino acid contents and the umami taste of the soy sauce peptide fraction SS−60%, the correlation of FAA contents with umami taste was analyzed using Pearson’s correlation analysis, as shown in Figure 5. There were strong positive correlations between the amino acid contents and the umami scores as well as the amino acid nitrogen contents of the soy sauce peptide fraction SS−60%. Particularly, it was found that the umami scores were significantly positively correlated with the contents of Phe, Glu, Ile, Leu, His, Tyr, Arg, Lys, Val, Met, Thr, Gly, and Ala as well as the UAAs and total FAAs (*p* < 0.05). Similar results were also observed for HAAs (Figure S3).

It was noted that, for FAAs, the umami scores did not show the highest correlation with Glu, the most recognized umami amino acid, compared with other amino acids. It might be due to the fact that on the one hand, the addition of sodium glutamate affected the original Glu level of soy sauce; on the other hand, not all soy sauces tested in this study had been added sodium glutamate. In contrast, the highest correlation was observed between the umami scores and Phe contents. Previous studies have revealed that Phe, sodium salt, and free Glu/MSG exhibited a strong umami taste [35]. In fact, Phe and Tyr, as L-α-aromatic amino acids, were important components of the flavor substances of soy sauce, and it had been confirmed that aromatic amino acids could enhance umami or salty taste [36].

**Figure 6:**
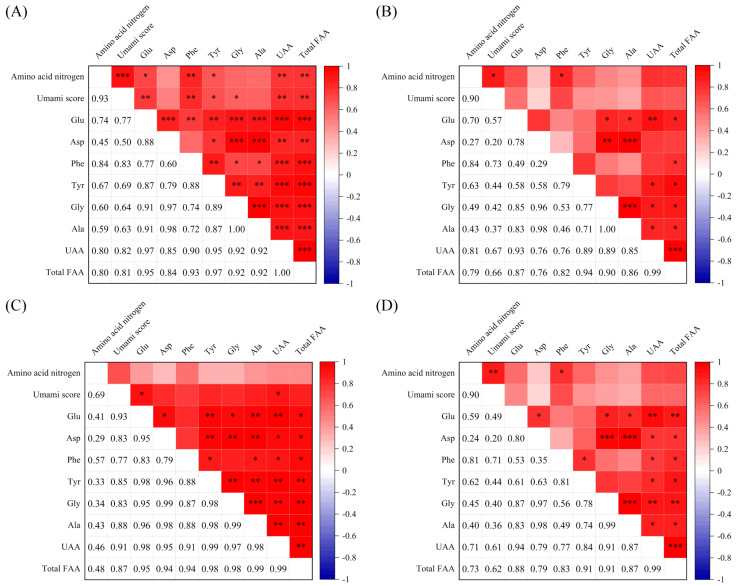
Pearson’s correlation analysis between the umami scores and free amino acid (FAA) contents of soy sauce peptide fraction SS−60%. (**A**) Ten soy sauces with added sodium glutamate; (**B**) six soy sauces with added sodium glutamate but not yeast extract. (**C**) Five soy sauces with added yeast extract; (**D**) seven soy sauces without yeast extract; UAAs: umami amino acids (Glu, Asp, Phe, Tyr, Ala, Gly). *** *p* < 0.001; ** *p* < 0.01; * *p* < 0.05.

The taste properties of soy sauce are affected by the raw materials used. For instance, sodium glutamate is usually used to improve the umami taste of soy sauce [37]. In this study, the correlation between the umami scores and the UAA contents of the FAAs of the peptide fraction SS−60% from ten soy sauces with added sodium glutamate was further analyzed and a relatively high correlation was observed compared with that of the peptide fraction SS−60% from 12 soy sauces, including 2 of them with sodium glutamate (Figure 6A). In addition, the yeast extract is rich in amino acids, peptides, and other substances with umami taste or umami-enhancing effects. As a consequence, yeast extract is also widely added directly to soy sauce to enhance the taste of the soy sauce [38]. In our present study, the umami scores of the peptide fraction SS−60% from six soy sauces without adding yeast extract showed lower correlations with the UAA contents (Figure 6B–D). These results confirmed that the addition of sodium glutamate and yeast extract altered the original UAA contents of soy sauces and made the correlations between the umami scores and UAA contents of the peptide fraction SS−60% more complex. However, no similar results were observed for HAAs (Figure S4). This suggested that the addition of sodium glutamate and yeast extract mainly altered the FAAs, not the HAAs.

### 3.5. The Mass Characteristics of Soy Sauce Peptide Fraction SS−60%

In our study, soy sauce peptide fraction SS−60% had a relatively higher contents of HAAs than FAAs, indicating that a portion of amino acids existed as peptides. Therefore, the potential peptide compositions of the soy sauce peptide fraction SS−60% from SS1, SS2, SS3, SS5, SS9, and SS10 were analyzed by MALDI−TOF MS.

**Figure 7:**
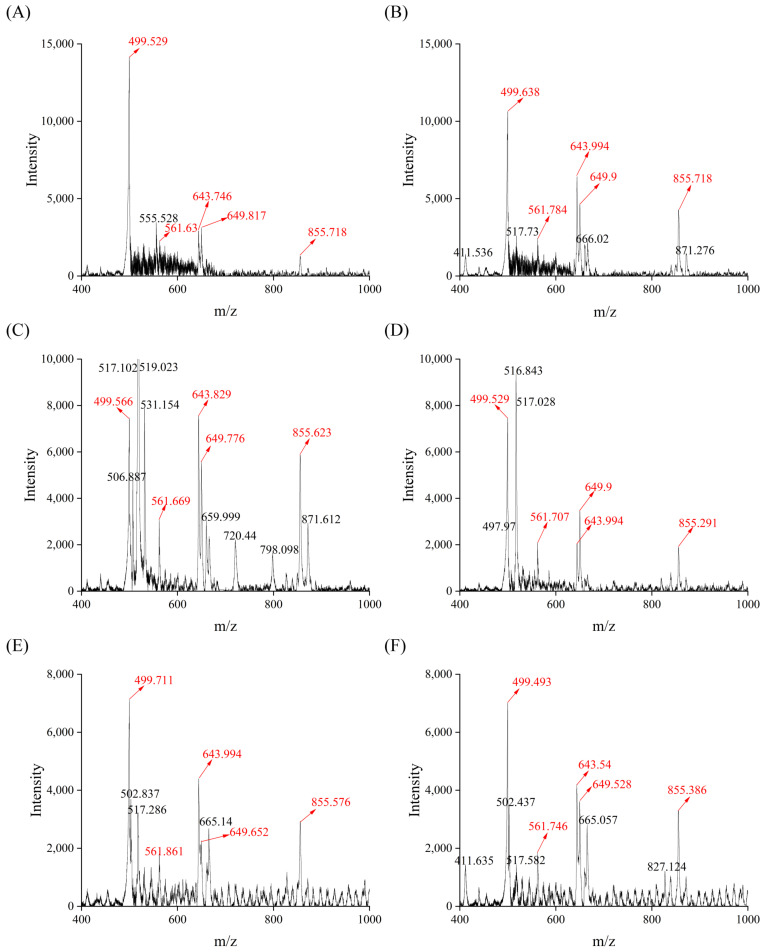
Mass spectra of soy sauce peptide fraction of soy sauce peptide fraction SS−60%. (**A**) SS1; (**B**) SS2; (**C**) SS3; (**D**) SS5; (**E**) SS9; (**F**) SS10.

In the present study, the *m*/*z* scan range was 400 to 5000, but the characteristic ion peaks of the soy sauce peptide fraction SS−60% mainly had *m*/*z* in the range of 400–1000. Meanwhile, the peptide fraction SS−60% from SS5, SS9, and SS10 showed a similar distribution of ion peaks with relatively low intensity within the *m*/*z* range of 1500–3500 (Figure S5). In order to evaluate the difference in soy sauce peptides more accurately, the characteristic ion peaks with *m*/*z* in the range of 400~1000 of the six soy sauce peptide fractions were analyzed and labeled as shown in Figure 7. It was found that these six soy sauce peptide fractions all had five noteworthy ion peaks with *m*/*z* at 499, 561, 643, 649, and 855, respectively. Among them, the ion peak with *m*/*z* at 499 showed the highest intensity and the intensity decreased with the amino acid contents of soy sauces. This indicated that these five ion peaks might be selected as the characteristic ion peaks in the peptide mass fingerprinting of soy sauce peptide fraction SS−60%. However, the peptide sequences of those characteristic ion peaks remain unknown and will be further identified in our next work.

## 4. Conclusions

This study found that the peptide fraction SS−60% isolated from soy sauce using XAD−16 macroporous resin adsorption combined with 60% ethanol elution showed a prominent umami taste, and the umami scores were highly positive correlated with the amino acid nitrogen contents of soy sauces. Amino acid composition analysis revealed that the amino acid contents of soy sauce peptide fraction SS−60% varied greatly, with Glu and Phe being the highest amino acids. The content of Phe showed the highest positive correlation with the umami scores. In contrast, the content of Glu had a relatively low correlation with the umami scores due to the addition of sodium glutamate and yeast extract, which had a great impact on the Glu contents and umami taste. Moreover, five characteristic ion peaks with *m*/*z* at 499, 561, 643, 649, and 855 were identified from the soy sauce peptide fraction SS−60% by MALDI−TOF MS. Overall, this study provides new insights into the umami characteristics of soy sauce with different amino acid nitrogen contents, showing potential usage in the real identification of soy sauce.

## Data Availability

The data that support the findings of this study are available from the corresponding author upon reasonable request.

## Supplementary Materials

The following supporting information can be downloaded at: https://www.mdpi.com/article/10.3390/foods13142242/s1, Table S1: Amino acid nitrogen content, production technology, and raw materials of soy sauce; Table S2: Free amino acid content of soy sauce peptide fractions eluted at 60% ethanol (results are expressed as mg/100 mg). Umami amino acids (Glu, Asp, Phe, Tyr, Ala, Gly): UAA.; Table S3: Hydrolyzed amino acid content of soy sauce peptide fractions eluted at 60% ethanol (results are expressed as mg/100 mg). Umami amino acids (Glu, Asp, Phe, Tyr, Ala, Gly): UAA; Table S4: Free amino acid accounted for the percentage of total amino acids. Umami amino acids (Glu, Asp, Phe, Tyr, Ala, Gly): UAA; Table S5: Hydrolyzed amino acid accounted for the percentage of total amino acids (%). Umami amino acids Glu, Asp, Phe, Tyr, Ala, Gly): UAA.; Figure S1: Taste characteristics of SS-0%, SS-20%, SS-40%, SS-60%. A: SS1; B: SS2; C: SS3; D: SS4; E: SS5; F: SS6; G: SS7; H: SS8; I: SS9; J: SS10; K: SS11; L: SS12; Figure S2: Umami score of SS-0%, SS-20%, SS-40%, SS-60%. A: SS1; B: SS2; C: SS3; D: SS4; E: SS5; F: SS6; G: SS7; H: SS8; I: SS9; J: SS10; K: SS11; L: SS12. Different letters indicate significant differences (*p* < 0.05); Figure S3: Pearson correlation analysis between the umami scores and hydrolyzed amino acid (HAA) contents of soy sauce peptide fraction SS-60%.UAA: umami amino acids (Glu, Asp, Phe, Tyr, Ala, Gly). ∗∗∗ *p* < 0.001; ∗∗ *p* < 0.01; ∗ *p*< 0.05; Figure S4: Pearson correlation analysis between the umami scores and hydrolyzed amino acid (HAA) contents of soy sauce peptide fraction SS-60%. A: Ten soy sauces added sodium glutamate; B: Six soy sauces added sodium glutamate but not yeast extract. C: Five soy sauces added yeast extract; D: Seven soy sauces without yeast extract; UAA: umami amino acids (Glu, Asp, Phe, Tyr, Ala, Gly). ∗∗∗ *p* < 0.001; ∗∗ *p* < 0.01; ∗ *p* < 0.05; Figure S5: Mass spectra of soy sauce peptide fraction of soy sauce peptide fraction SS-60%. A: SS1; B: SS2; C: SS3; D: SS5; E: SS9; F: SS10.
