# Supplementary material for: Characteristics of Umami Taste of Soy Sauce Using Electronic Tongue, Amino Acid Analyzer, and MALDI−TOF MS
Source: Foods. 2024 Jul 16;13(14):2242. doi: 10.3390/foods13142242 (PMC11276169; doi:10.3390/foods13142242)
Supplement: Supplementary file 1 [file foods-13-02242-s001.zip › foods-3072452-supplementary.pdf]

Table S1. Amino acid nitrogen content, production technology and raw materials of soy sauce

Type	Amino Acid Nitrogen Content (G/100 Ml)	Production Technology	Raw Material
SS1	1.25	High salt dilute fermentation	Water, Soya beans, Salt, Wheat, Sugar, Monosodium glutamate, Disodium 5'−ribonucleotide, Disodium 5'−−inosinate, Sucralose, High fructose syrup
SS2	1.2	High salt dilute fermentation	Water, Soya beans, Salt, Wheat, Monosodium glutamate, Sugar, Disodium 5'−−inosinate, Disodium 5'−−ribonucleotide, Yeast extract, Sucralose
SS3	1.2	High salt dilute fermentation	Water, Soya beans, Salt, Wheat, Sugar, Yeast extract, Monosodium glutamate, Disodium 5'−−ribonucleotide, Disodium 5'−−inosinate, Sucralose
SS4	1	High salt dilute fermentation	Water, Soya beans, Salt, Wheat, Sugar, Yeast extract
SS5	1	High salt dilute fermentation	Water, Soya beans, Salt, Wheat, Sugar, Rice, Yeast extract, Monosodium glutamate, Disodium 5'−−ribonucleotide, Disodium 5'−−inosinate, Potassium sorbate, Sucralose
SS6	1	High salt dilute fermentation	Water, Soya beans, Salt, Wheat, Monosodium glutamate, Sugar, Rice, Yeast extract, Disodium 5'−−ribonucleotide, Disodium 5'−−inosinate, Potassium sorbate, Sucralose
SS7	0.8	High salt dilute fermentation	Water, Soya beans, Salt, Wheat, Monosodium glutamate, Sugar, Disodium 5'−−ribonucleotide, Disodium 5'−−inosinate, Scallop, Sucralose
SS8	0.7	High salt dilute fermentation	Water, Soya beans, Salt, Sugar, Wheat, Straw mushroom
SS9	0.7	High salt dilute fermentation	Water, Soya beans, Caramel color, Sugar, Salt, Wheat, Wheat flour, Monosodium glutamate, Disodium 5'−−ribonucleotide, Disodium 5'−−inosinate

Continued Table S1.

**Type**	**Amino Acid Nitrogen Content(G/100ml)**	**Production Technology**	**Raw Material**
SS10	0.5	High salt dilute fermentation	Water, Soya beans, Salt, Wheat, Monosodium glutamate, Wheat flour, High fructose syrup, Rice, Disodium 5’−−ribonucleotide, Disodium 5’−−inosinate, Caramel color, Potassium sorbate, Sucralose
SS11	0.4	High salt dilute fermentation	Water, Soya beans, Salt, Wheat, Monosodium glutamate, Wheat flour, High fructose syrup, Disodium 5’−−ribonucleotide, Disodium 5’−−inosinate, Caramel color, Potassium sorbate, Sucralose
SS12	0.4	High salt dilute fermentation	Water, Soya beans, Salt, Caramel color, Wheat, Monosodium glutamate, Wheat flour, Disodium 5’−−ribonucleotide, Disodium 5’−−inosinate, Potassium sorbate, Sucralose

Table S2. Free amino acid content of soy sauce peptide fractions eluted at 60% ethanol (results are expressed as mg/100 mg). Umami amino acids (Glu, Asp, Phe, Tyr, Ala, Gly): UAA.

	Free Amino Acid (mg/100 mg)											
**Amino Acid**	**SS1**	**SS2**	**SS3**	**SS4**	**SS5**	**SS6**	**SS7**	**SS8**	**SS9**	**SS10**	**SS11**	**SS12**
Glu	1.063	1.334	1.515	0.255	0.717	1.860	0.616	0.034	0.934	0.908	0.257	0.033
Asp	0.121	0.170	0.194	0.084	0.078	0.262	0.118	0.038	0.112	0.231	0.031	0.014
Val	0.107	0.157	0.160	0.072	0.063	0.174	0.092	0.017	0.056	0.139	0.027	0.008
Met	0.020	0.046	0.022	0.027	0.018	0.036	0.030	0.024	——	0.025	0.007	——
Ile	0.163	0.252	0.207	0.104	0.114	0.256	0.160	0.053	0.062	0.136	0.040	0.017
Leu	0.282	0.426	0.335	0.168	0.186	0.427	0.259	0.079	0.101	0.209	0.067	0.028
Phe	0.683	1.116	0.735	0.371	0.447	0.963	0.647	0.125	0.096	0.195	0.131	0.028
His	0.288	0.468	0.334	0.480	0.299	0.407	0.303	0.165	0.103	0.077	0.150	0.068
Tyr	0.047	0.064	0.059	0.030	0.040	0.080	0.054	0.027	0.031	0.042	0.015	——
Arg	0.052	0.076	0.067	0.009	0.010	0.071	0.014	0.029	0.020	0.014	——	——
Lys	0.150	0.316	0.232	0.135	0.111	0.271	0.173	0.045	0.073	0.161	0.050	0.019
Pro	0.124	0.181	0.142	0.035	0.099	0.227	0.157	0.118	0.095	0.129	0.040	0.021
Thr	0.066	0.095	0.089	0.061	0.042	0.113	0.061	0.018	0.042	0.091	0.016	0.007
Ser	0.091	0.121	0.124	0.248	0.058	0.160	0.080	0.029	0.063	0.133	0.022	0.011
Gly	0.057	0.072	0.069	0.038	0.037	0.093	0.050	0.014	0.038	0.076	0.014	0.007
Ala	0.084	0.117	0.116	0.053	0.053	0.146	0.072	0.027	0.057	0.124	0.018	0.009
UAA	2.056	2.872	2.690	0.830	1.371	3.403	1.556	0.266	1.268	1.575	0.466	0.091
Total FAA	3.398	5.010	4.403	2.169	2.372	5.544	2.887	0.843	1.884	2.690	0.885	0.270

——: not detected

Table S3 Hydrolyzed amino acid content of soy sauce peptide fractions eluted at 60% ethanol (results are expressed as mg/100 mg). Umami amino acids (Glu, Asp, Phe, Tyr, Ala, Gly): UAA.

	Hydrolyzed Amino Acid (mg/100 mg)											
**Amino Acid**	**SS1**	**SS2**	**SS3**	**SS4**	**SS5**	**SS6**	**SS7**	**SS8**	**SS9**	**SS10**	**SS11**	**SS12**
Glu	1.758	1.744	1.659	0.996	0.755	2.552	1.237	0.307	1.131	1.137	0.675	0.272
Asp	0.543	0.768	0.521	1.034	0.347	0.696	0.291	0.229	0.153	0.420	0.260	0.114
Val	0.385	0.504	0.323	0.530	0.240	0.469	0.413	0.134	0.116	0.229	0.188	0.143
Met	0.095	0.132	0.095	0.153	0.054	0.122	——	0.030	——	0.051	0.054	——
Ile	0.418	0.574	0.358	0.630	0.244	0.568	0.579	0.216	0.102	0.189	0.159	0.180
Leu	0.608	0.832	0.526	0.898	0.379	0.841	0.794	0.287	0.147	0.303	0.233	0.246
Phe	0.959	1.289	0.771	1.216	0.506	1.420	1.258	0.357	0.172	0.401	0.065	0.315
His	0.527	0.670	0.530	0.600	0.374	0.613	0.482	0.060	0.079	0.084	0.077	0.112
Tyr	0.201	0.372	0.216	0.412	0.157	0.246	0.180	0.077	0.059	0.105	——	0.046
Arg	0.147	0.198	0.094	0.358	0.037	0.236	0.169	0.935	0.039	0.065	0.041	0.066

Lys	0.442	0.581	0.403	0.705	0.264	0.506	0.505	0.058	0.071	0.193	0.141	0.151
Pro	0.533	1.045	0.609	1.228	0.439	0.685	0.891	0.120	0.120	0.378	0.296	0.286
Thr	0.348	0.392	0.239	0.418	0.162	0.316	0.152	0.100	0.052	0.176	0.118	0.049
Ser	0.278	0.401	0.268	0.414	0.173	0.353	0.307	0.110	0.096	0.206	0.123	0.047
Gly	0.387	0.459	0.302	0.743	0.214	0.395	0.508	0.149	0.067	0.183	0.148	0.173
Ala	0.186	0.235	0.175	0.463	0.105	0.252	0.281	0.068	0.058	0.142	0.157	0.097
UAA	4.034	4.867	3.643	4.864	2.086	5.561	3.753	1.188	1.640	2.388	1.306	1.018
Total HAA	7.814	10.196	7.089	10.799	4.452	10.270	8.045	3.237	2.460	4.261	2.735	2.299

——: not detected

Table S4. Free amino acid accounted for the percentage of total amino acids. Umami amino acids (Glu, Asp, Phe, Tyr, Ala, Gly): UAA.

	**Free Amino Acid Accounted for the Percentage of Total Amino Acids (%)**											
**Amino Acid**	**SS1**	**SS2**	**SS3**	**Ss4**	**Ss5**	**Ss6**	**Ss7**	**Ss8**	**Ss9**	**Ss10**	**Ss11**	**Ss12**
Glu	31.28	26.62	34.42	11.75	30.22	33.54	21.32	4.06	49.58	33.74	29.10	12.16
Asp	3.55	3.40	4.41	3.85	3.28	4.73	4.09	4.53	5.92	8.61	3.48	5.23
Val	3.15	3.13	3.63	3.32	2.67	3.13	3.20	2.02	3.00	5.17	3.05	2.91
Met	0.58	0.92	0.50	1.23	0.77	0.66	1.04	2.84	0.00	0.92	0.81	0.00
Ile	4.80	5.03	4.70	4.80	4.79	4.61	5.53	6.29	3.32	5.07	4.49	6.19
Leu	8.30	8.51	7.61	7.76	7.85	7.71	8.99	9.37	5.34	7.76	7.60	10.47
Phe	20.11	22.27	16.70	17.09	18.86	17.38	22.41	14.82	5.12	7.23	14.78	10.29
His	8.47	9.33	7.59	22.12	12.60	7.34	10.51	19.55	5.48	2.85	16.91	25.06
Tyr	1.40	1.27	1.35	1.40	1.67	1.44	1.87	3.19	1.63	1.57	1.65	0.00
Arg	1.52	1.51	1.53	0.42	0.44	1.29	0.50	3.45	1.07	0.53	0.00	0.00
Lys	4.42	6.31	5.27	6.21	4.67	4.88	6.01	5.30	3.89	5.99	5.64	7.07
Pro	3.66	3.61	3.23	1.59	4.19	4.09	5.44	13.98	5.03	4.81	4.53	7.91
Thr	1.93	1.90	2.03	2.81	1.75	2.03	2.11	2.19	2.25	3.39	1.79	2.72
Ser	2.69	2.41	2.81	11.43	2.46	2.88	2.77	3.48	3.34	4.94	2.52	3.94
Gly	1.69	1.44	1.57	1.76	1.54	1.67	1.73	1.71	2.02	2.82	1.57	2.62
Ala	2.47	2.33	2.64	2.45	2.24	2.63	2.50	3.21	3.02	4.59	2.07	3.42
UAA	60.50	57.33	61.09	38.29	57.82	61.39	53.92	31.52	67.29	58.56	52.66	33.73

Table S5. Hydrolyzed amino acid accounted for the percentage of total amino acids (%). Umami amino acids Glu, Asp, Phe, Tyr, Ala, Gly): UAA.

	Hydrolyzed Amino Acid Accounted for the Percentage of Total Amino Acids (%)											
**Amino Acid**	**SS1**	**SS2**	**SS3**	**SS4**	**SS5**	**SS6**	**SS7**	**SS8**	**SS9**	**SS10**	**SS11**	**SS12**
Glu	22.50	17.10	23.40	9.22	16.96	24.85	15.37	9.49	45.96	26.68	24.67	11.85
Asp	6.95	7.53	7.35	9.58	7.79	6.78	3.61	7.06	6.21	9.86	9.51	4.97
Val	4.92	4.95	4.56	4.91	5.38	4.56	5.14	4.13	4.70	5.37	6.86	6.21
Met	1.21	1.29	1.34	1.42	1.22	1.19	0.00	0.93	0.00	1.19	1.98	0.00
Ile	5.36	5.63	5.06	5.83	5.49	5.53	7.19	6.68	4.14	4.44	5.80	7.81
Leu	7.78	8.16	7.42	8.32	8.52	8.19	9.87	8.87	5.96	7.11	8.54	10.71
Phe	12.27	12.64	10.88	11.26	11.38	13.82	15.63	11.03	6.97	9.41	2.39	13.71
His	6.74	6.58	7.48	5.56	8.40	5.97	6.00	1.84	3.20	1.97	2.81	4.87
Tyr	2.57	3.65	3.05	3.81	3.53	2.40	2.24	2.39	2.42	2.46	0.00	2.01
Arg	1.88	1.94	1.32	3.32	0.84	2.29	2.10	28.89	1.57	1.53	1.49	2.88
Lys	5.65	5.70	5.69	6.53	5.94	4.93	6.28	1.79	2.89	4.53	5.14	6.57
Pro	6.82	10.25	8.59	11.37	9.85	6.67	11.08	3.70	4.86	8.86	10.83	12.45
Thr	4.46	3.84	3.38	3.87	3.63	3.08	1.88	3.08	2.12	4.12	4.33	2.15
Ser	3.55	3.93	3.78	3.83	3.88	3.44	3.82	3.41	3.90	4.83	4.49	2.06
Gly	4.95	4.50	4.26	6.88	4.82	3.85	6.32	4.61	2.74	4.30	5.41	7.54
Ala	2.38	2.31	2.47	4.29	2.37	2.46	3.49	2.10	2.36	3.34	5.76	4.20
UAA	51.62	47.73	51.40	45.04	46.84	54.14	46.66	36.69	66.66	56.04	47.73	44.28

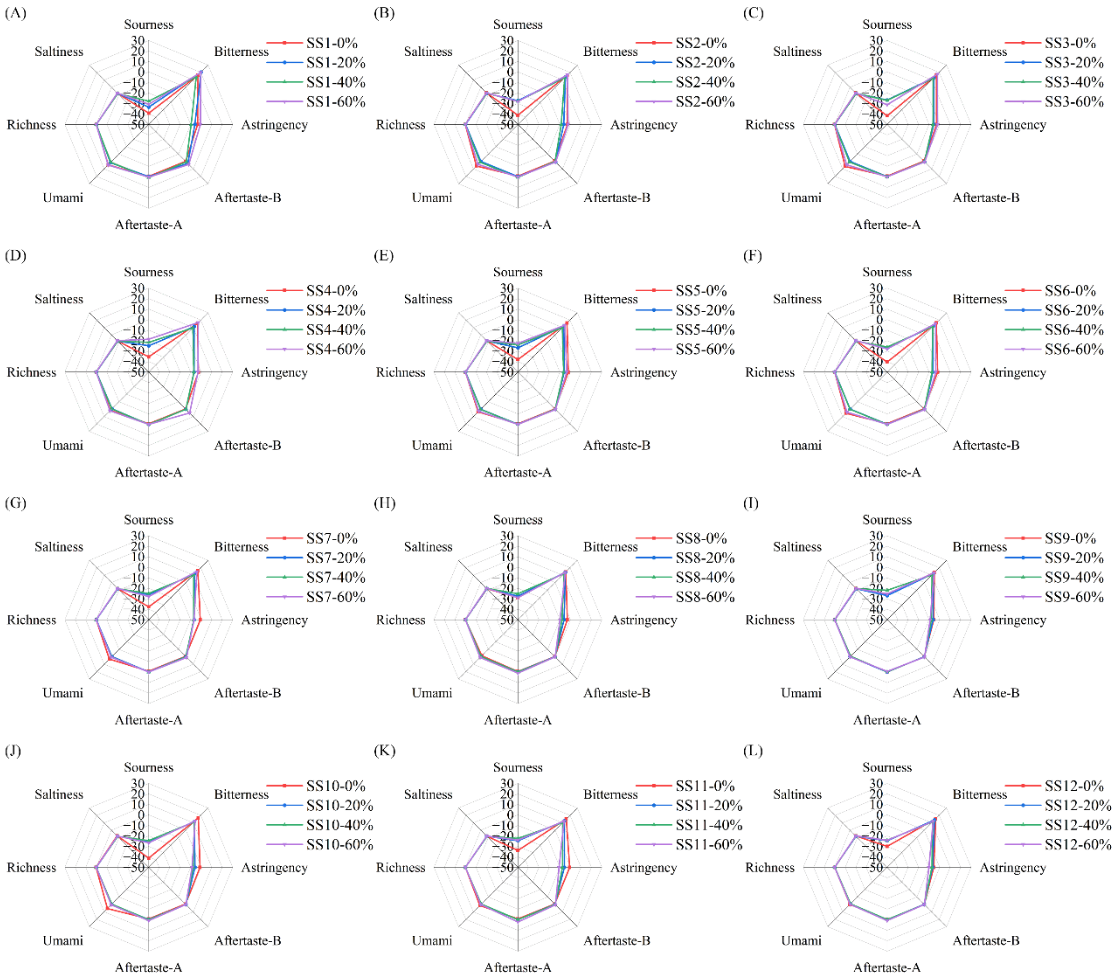

Figure S1. Taste characteristics of SS−0%, SS−20%, SS−40%, SS−60%. A: SS1; B: SS2; C: SS3; D: SS4; E: SS5; F: SS6; G: SS7; H: SS8; I: SS9; J: SS10; K: SS11; L: SS12

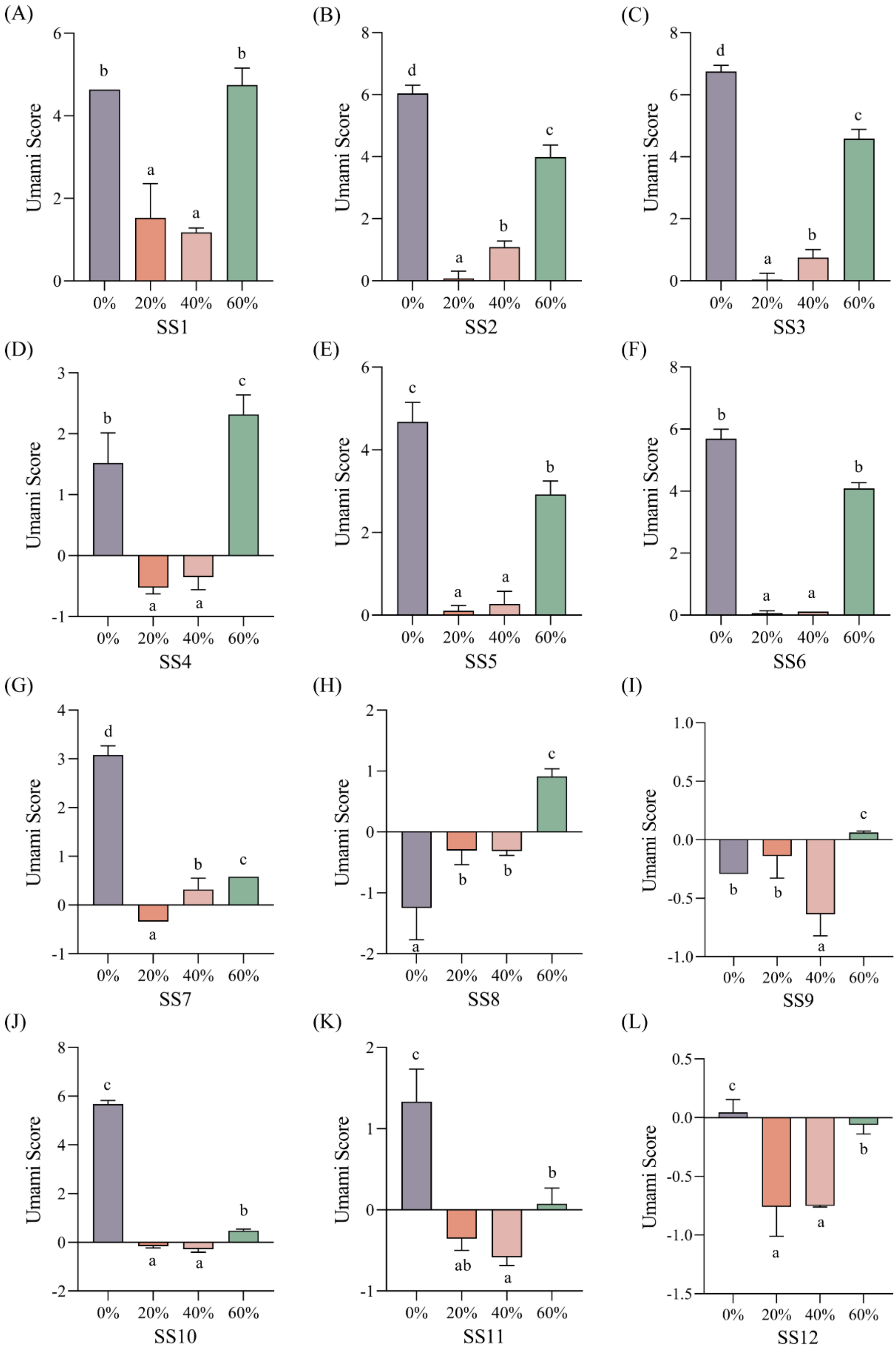

Figure S2. Umami score of SS−0%, SS−20%, SS−40%, SS−60%. A: SS1; B: SS2; C: SS3; D: SS4; E: SS5; F: SS6; G: SS7; H: SS8; I: SS9; J: SS10; K: SS11; L: SS12. Different letters indicate significant differences (p< 0.05).

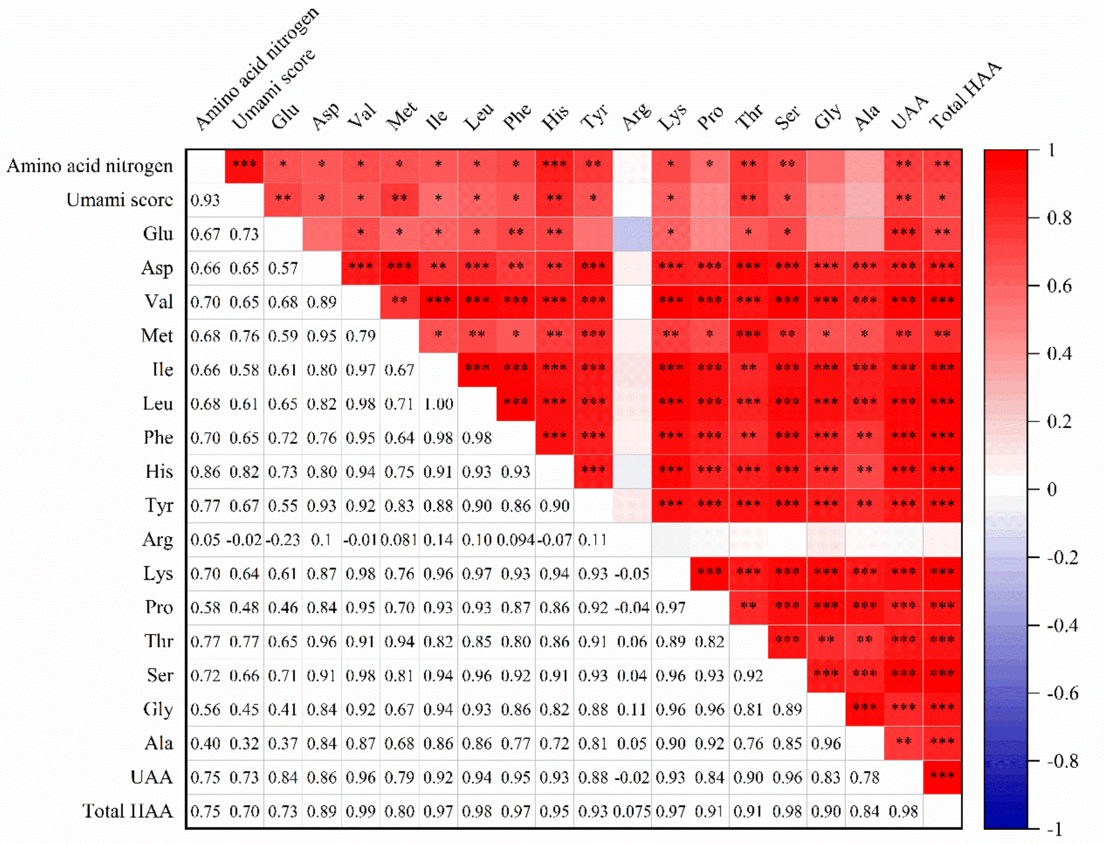

Figure S3. Pearson correlation analysis between the umami scores and hydrolyzed amino acid (HAA) contents of soy sauce peptide fraction SS−60%. UAA: umami amino acids (Glu, Asp, Phe, Tyr, Ala, Gly). *** $p < 0.001$; ** $p < 0.01$; * $p < 0.05$

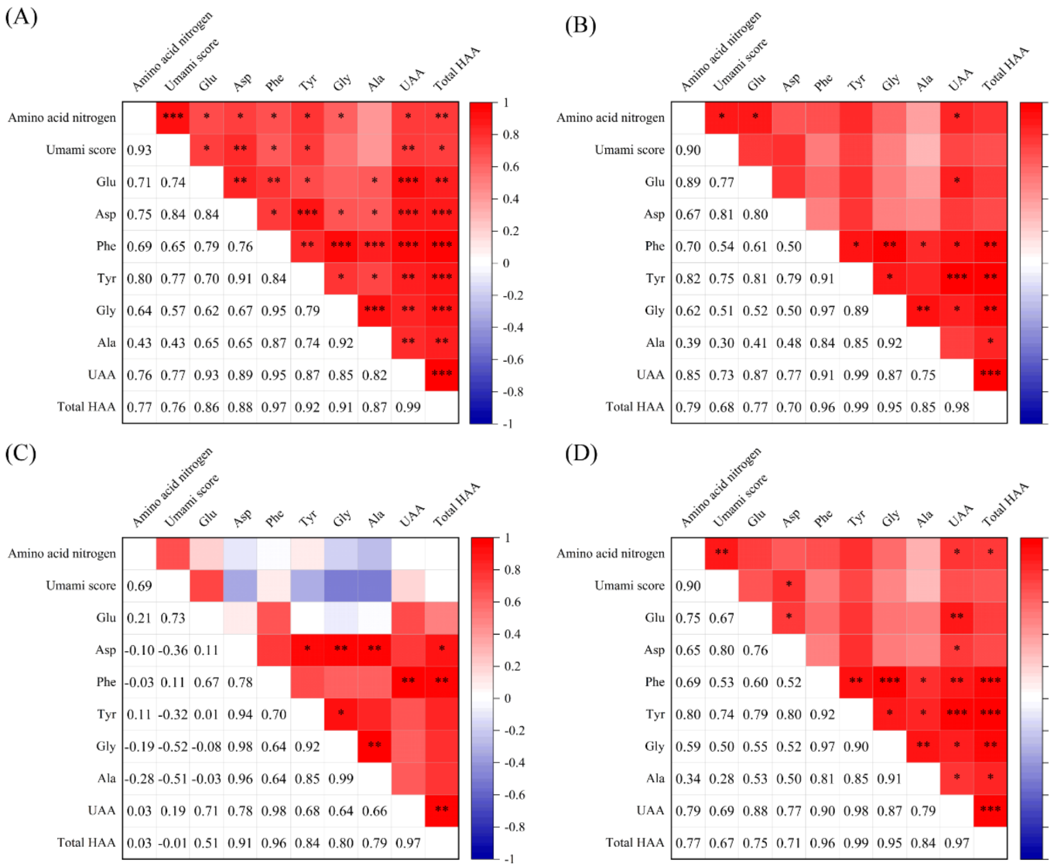

Figure S4. Pearson correlation analysis between the umami scores and hydrolyzed amino acid (HAA) contents of soy sauce peptide fraction SS−60%. A: Ten soy sauces added sodium glutamate; B: Six soy sauces added sodium glutamate but not yeast extract. C: Five soy sauces added yeast extract; D: Seven soy sauces without yeast extract; UAA: umami amino acids (Glu, Asp, Phe, Tyr, Ala, Gly). *** $p < 0.001$; ** $p < 0.01$; * $p < 0.05$

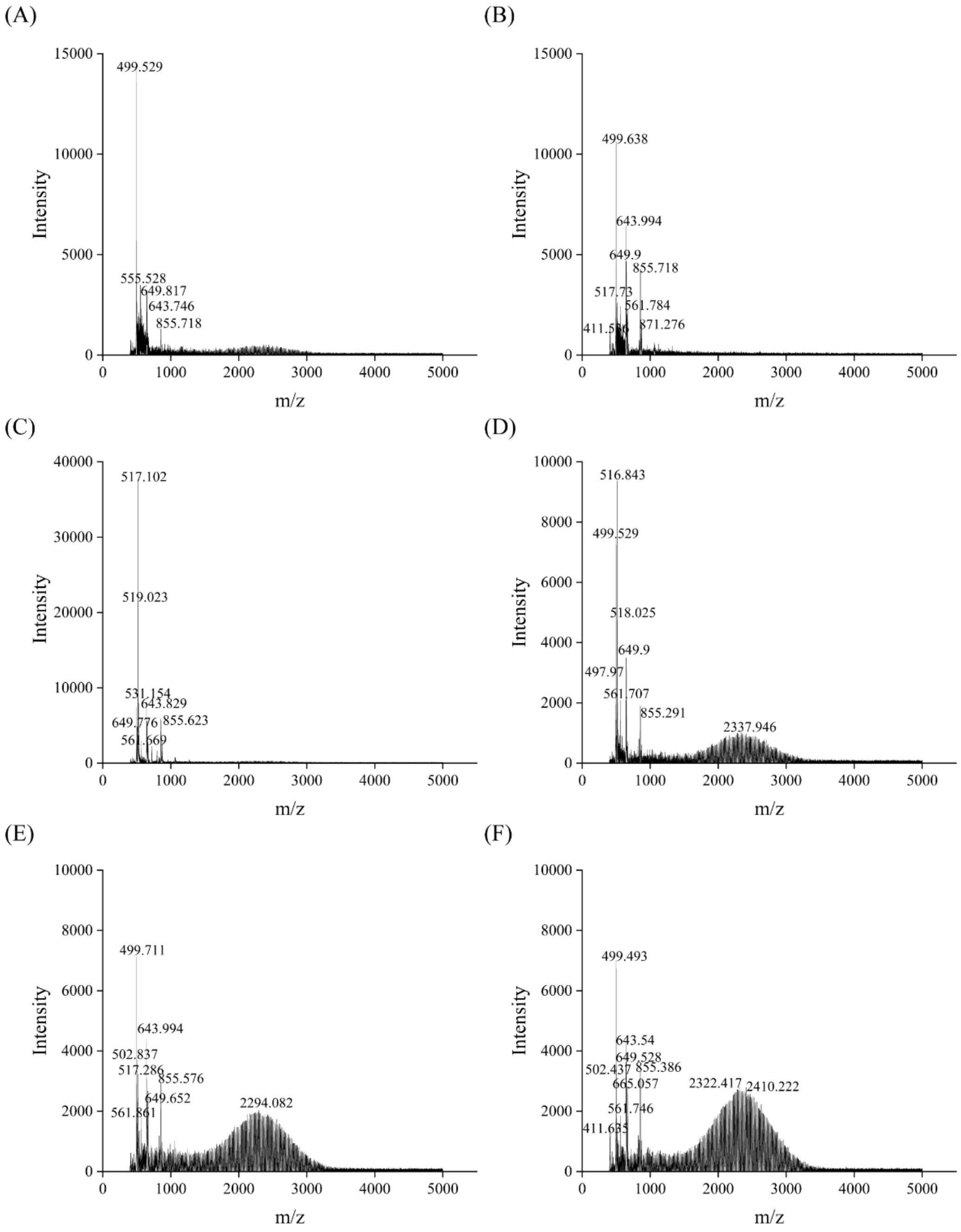

Figure S5. Mass spectra of soy sauce peptide fraction of soy sauce peptide fraction SS−60%. A: SS1; B: SS2; C: SS3; D: SS5; E: SS9; F: SS10.
